# Prevalence of Food Insecurity and Associations with Academic Performance, Food Consumption and Social Support among University Students during the COVID-19 Pandemic: FINESCOP Project in Iceland

**DOI:** 10.3390/nu16060764

**Published:** 2024-03-07

**Authors:** Brittany M. Repella, James G. Rice, Marta Arroyo-Izaga, Liv E. Torheim, Bryndis E. Birgisdottir, Greta Jakobsdottir

**Affiliations:** 1 Faculty of Health Promotion, Sport and Leisure Studies, School of Education, University of Iceland, 105 Reykjavik, Iceland; gretaja@hi.is; 2 Faculty of Sociology, Anthropology and Folkloristics, School of Social Sciences, University of Iceland, 101 Reykjavik, Iceland; james@hi.is; 3 BIOMICs Research Group, Microfluidics & BIOMICs Cluster, Department of Pharmacy and Food Sciences, Lascaray Research Center, University of the Basque Country UPV/EHU, Bioaraba, BA04.03, 01006 Vitoria-Gasteiz, Spain; marta.arroyo@ehu.eus; 4 Department of Nursing and Health Promotion, Faculty of Health Sciences, Oslo Metropolitan University, 0176 Oslo, Norway; livtor@oslomet.no; 5 Faculty of Food Science and Nutrition, School of Health Sciences, University of Iceland, 102 Reykjavik, Iceland; beb@hi.is

**Keywords:** food security, university students, coronavirus, academic performance, food consumption

## Abstract

(1) Background: Food insecurity (FI) among university students has received less attention in Europe than in other regions before and during the COVID-19 pandemic. (2) Methods: A cross-sectional study was conducted between January and March 2022 using an online questionnaire (*n* = 924). The questionnaire addressed food security status; demographic, socioeconomic, and educational variables; academic performance; food consumption; and social support networks. The validated Food Insecurity Experience Scale was used to measure food security. Binary logistic regressions adjusted by age and gender were applied to identify FI-related factors. (3) Results: Just over 17% of the students were living with some level of FI, nearly one in three students reported having consumed few kinds of food, and 3.9% spent an entire day without eating due to a lack of resources. Food insecurity was associated with a higher likelihood of negative academic performance, decreased food consumption, and a lower likelihood of having a large support network, when compared to food-secure respondents. (4) Conclusions: The findings suggest that FI negatively impacts students’ academic performance and food consumption. Future public health programs should be prioritized to prevent students from experiencing hunger due to financial or resource constraints.

## 1. Introduction

The United Nations Food and Agriculture Organization (FAO) defines food insecurity (FI) as limited access to readily available, nutritionally suitable, and safe foods for all [1,2]. A person’s food security status and socioeconomic standing greatly depend on income, which can refer to running out of food, being unable to afford enough food, or having poorer diet quality due to limited money, among others [3]. This definition can be used for all groups, including university students or young adults. The economic status of the general population in Iceland is relatively good; however, the gap between the richest and poorest continues to widen, with nearly 9% of the population considered poor, according to Statistics Iceland [4].

University students are at higher risk for FI compared to the general adult population. Recent papers from the US show the average prevalence of FI among US university students ranges from 19 to 34% [5,6,7] and in other countries, for example, from 11 to 42% as seen in other research [8,9,10]. While the main reason why university students are at high risk for FI is unclear, it can be hypothesized that their young age, employment or income level, housing situation, and financial aid or loan status may be relevant factors [11,12]. In Iceland, financial aid or student loans are available to all students and are typically used to cover living costs rather than tuition fees. Yearly tuition fees for higher education at public universities (such as the University of Iceland and the University of Akureyri) are approximately EUR 500. For private universities (such as Reykjavik University), the tuition is approximately EUR 5000 per year, divided into two semesters. This is considered highly affordable compared to many other countries.

With COVID-19, new and more efficient methods are needed to support university students, including strategies to prevent FI or hunger. As an illustration of the impact of the pandemic on student well-being, the Hope Center for College, Community, and Justice performed a survey of undergraduate students among 54 colleges and universities in 26 states in the US, which included 15 four-year (undergraduate and graduate schools) and 39 two-year (community college) establishments. Results found that 38% of students from a four-year university or college and 44% from a two-year college reported FI over the previous 30 days [13]. Such data underscore the pressing need for targeted interventions and support systems to alleviate food insecurity among university students during these challenging times.

In recent years, students’ ability to cope with the COVID-19 pandemic may have also affected their food security status. An early report by Owens et al. found that FI among university students has increased from the pre-pandemic level [5]. Some factors that may be to blame for lower food security among students include higher unemployment rates; increased costs of goods due to inflation; the need to move away from on-campus housing when schools shut down; closures of campus dining halls, which may affect students with low food skills or a low ability to shop, prepare, and cook nutritious meals more severely; and the limited access to or eligibility for community resources [13,14,15]. When examining the characteristics of students in Iceland, they tend to be older than those in many other countries and more commonly work alongside their studies, often while also raising children. According to the survey “Eurostudent” conducted from 2018 to 21, 59% of students in Iceland were older than 25 years, compared to 32% of students from other European countries participating in the survey. This survey covers 26 European countries, examining students’ social and economic conditions. Additionally, 33% of Icelandic students have children, compared to 16% and 10% in Scandinavian countries and Europe, respectively. Regarding the number of students working during their studies, 68% of Icelandic students have a job compared to 51% of average European students [16].

In any case, within European countries, the issue of FI among college and university students has not received as much focus compared to other regions globally, both before the pandemic [17] and in the context of the pandemic [18]. To address this gap in the literature, the present research, conducted from January to March 2022, aims to analyze the prevalence of FI and possible related factors among university students in Iceland during the COVID-19 pandemic. Specifically, the related factors that were analyzed included changes in academic performance, the amount of food consumed during the pandemic, and social support networks. The results of this research may help with policy development and creation aimed at reducing FI among university students with consideration of the COVID-19 pandemic.

## 2. Materials and Methods

### 2.1. Study Design

This study is part of the research project Food Insecurity among European University Students during the COVID-19 Pandemic (FINESCOP). FINESCOP is a cross-sectional investigation with the primary objective of understanding the challenging and vulnerable situation among university students around Europe, especially during the COVID-19 pandemic, in addition to demands on academic performance and other aspects related to health and lifestyle. Other colleges and universities participating in FINESCOP throughout Europe included Norway, Finland, Germany, Poland, Netherlands, Belgium, Portugal, Spain, and Italy. However, the current dissemination will refer to the Icelandic results only.

### 2.2. Participant Recruitment and Enrollment

The questionnaire was administered online via a Qualtrics survey software (Provo, UT, USA, available at: www.qualtrics.com/ (accessed on 1 November 2021)) from 11 January 2022 to 31 March 2022, at three of the largest universities in Iceland: the University of Iceland (public), the University of Akureyri (public), and Reykjavik University (private). Eligibility criteria included being a matriculated student of one of Iceland’s three participating universities and 18 years or older. Students were required to have access to their university email.

The student registration office shared the survey link via email with approximately 20,800 students. Two emails containing the link to the questionnaire were sent, as well as a reminder email. Flyers advertising the study with a QR code were also hung up around campus. The participants were digitally informed about the purpose of the project and the usage and storage of the collected data and were digitally asked for consent before starting the questionnaire.

The online questionnaire was designed to be completed in approximately 20 min on a computer or mobile device. Nine hundred and twenty-four completed the survey (response rate: 4.4%). All responses were kept, even if respondents left some questions blank. However, and only for the food security status analysis, 197 were removed from the categorization due to incomplete data, which would have compromised the accuracy of categorization based on the Food Insecurity Experience Scale [19].

### 2.3. Development of the Questionnaire

The questionnaire was developed in English through collaboration with all the partners in the FINESCOP consortium, all of whom participated in the selection and consensus of the measurement variables and tools [8]. A three-stage pilot of the questionnaire was carried out by five of the eleven universities participating in the project: (1) initial development of the questionnaire, (2) structured testing in the field, and (3) practical implementation. During the first phase, a preliminary version of the questionnaire was created, available in both English and Icelandic, the local language, using a rigorous parallel and back translation method to ensure linguistic precision. The second phase of structured piloting adhered to the guidelines outlined in the Development Impact Evaluation’s survey piloting guide [20]. The final stage was a field implementation practice, engaging a cohort of Icelandic students (*n* = 15). This final stage provided invaluable insights into the questionnaire’s applicability and effectiveness in Icelandic. Further insights into the development of the FINESCOP questionnaire can be seen in the publication by González-Pérez and colleagues, which includes information on the reliability index for the socioeconomic and educational sections in Spain, which resulted in Cronbach’s α being 0.83 and 0.79, respectively [8].

The questions used to define individual food security were taken from the Food and Agriculture Organization Food Insecurity Experience Scale (FIES) [19]. The questions refer to the last 12 months. The responses include “yes”, “no”, “unsure”, and “do not want to answer”.

In addition, the compulsory questionnaire included the following demographic variables: participant’s age, gender, birthplace, and migration status (if applicable); socioeconomic variables: employment, income, living arrangements, participation in food assistance programs or other types of food assistance, and parents’ educational level; educational variables: campus, their field of education, study level, academic year, teaching modality, and scholarships; and weight status. All the questions related to demographic, socioeconomic, and educational variables were taken from the questionnaires developed and used by Owens et al. [5] and Mahdy [21], except the questions related to migration status [22] and parents’ educational level [23]. An additional question on support systems was added and was not standard on the FINESCOP questionnaires. This was added to support comparison with another survey conducted in Iceland.

### 2.4. Ethical Consideration

This study followed ethical standards to ensure the protection of respondents. All respondents completed a digital consent form before participating in the survey and were only allowed to participate once consent was received. This study was reviewed and approved by the institutional review board of the university’s Ethics Committee for Scientific Research (SHV2021-038).

### 2.5. Data Analysis

Descriptive and comparative statistics were analyzed within the Qualtrics survey software (Provo, UT, USA) and in RStudio v4.6.4 (R Core Team 2022). Some variables were grouped when appropriate to facilitate the analysis and make comparisons. Rosner’s Outlier Test and histograms created in RStudio were used to check the distribution among variables and to test for outliers in numerical variables.

A modified version of the USDA’s scoring system was utilized to classify respondents as food-secure (zero to two “yes” responses) or food-insecure (three to eight “yes” responses) by using the raw score of food security indicators [23]. In addition, the FIES variable was categorized according to the criteria of Ballard et al.: food-secure (total raw score of 0) and food-insecure (total raw score of 1–8) [1]. While the categorization from Ballard et al. was used for comparison with other FINESCOP analyses [8], the USDA’s scoring system was used here to compare to all other results in the current paper. Specific food security indicators left empty were considered incomplete for categorization and, therefore, excluded from the food security analysis. Of the 924 participants, 197 cases that did not finish all the FIES items were excluded. Thus, 727 remained in the data set. Those that had the responses of “unsure” and “do not want to answer” were given a value of 0 [24].

Regression analysis adjusted by gender and age was used to identify the FI-related factors. The results are presented as an odds ratio (OR) with a 95% confidence interval (CI). The following dependent variables were included in this analysis: changes in academic performance, the amount of food consumed during the pandemic, and social support networks. The reference categories were those reported in the data analysis to be food-secure. *p*-values less than 0.05 were considered statistically significant.

### 2.6. Recategorization of Variable Responses

The responses for the variable “academic performance” were grouped and renamed as the following: positive performance (“in a very positive way” and “in a somewhat positive way”), negative performance (“in a very negative way” and “in a somewhat negative way”), neutral performance (“not affected”), and do not know (“don’t know or prefer not to say”).

## 3. Results

### 3.1. Respondent Characteristics

The sample’s demographic, socioeconomic, and educational characteristics are in Table 1. The respondents were primarily female (74.5%), with origins in Iceland (78.5%), and most were residing in the capital region at the time of the study (75.1%). The average age of the respondents was 31.7 years (SD 8.4). Many respondents were employed full- or part-time before COVID-19 (30.7% and 33.9%, respectively). It was not specified if students in Ph.D. programs receiving student funding for their education considered this employment. When asked about the highest level of education achieved by a parent or legal guardian, most respondents’ parents or guardians had received their first (BS or BA) or second (MSc or Ph.D.) stage of tertiary education (26.5% and 33.9%, respectively).

Over half of the respondents were undergraduates (53.1%) and either in their first or second year of studies (28.2% and 29%, respectively). While their fields of study were mixed, most respondents were currently studying within the field of health and wellness (18.4%). With COVID-19 restrictions occurring at the time of the survey, more than half of the students received a blended type of teaching, online and face-to-face on campus (51%), as seen in Table 2.

### 3.2. Prevalence of FI during the COVID-19 Pandemic

Applying the criteria of the modified version of the USDA’s scoring system [22], the resulting proportion of the sample population experiencing FI was 17% (*n* = 125), and the rest was categorized as food-secure (83%) (*p* < 0.001). Meanwhile, according to the criteria of Ballard et al. [1], 29.8% were considered food-insecure and 70.2% as food-secure (*p* < 0.001).

**Figure 1:**
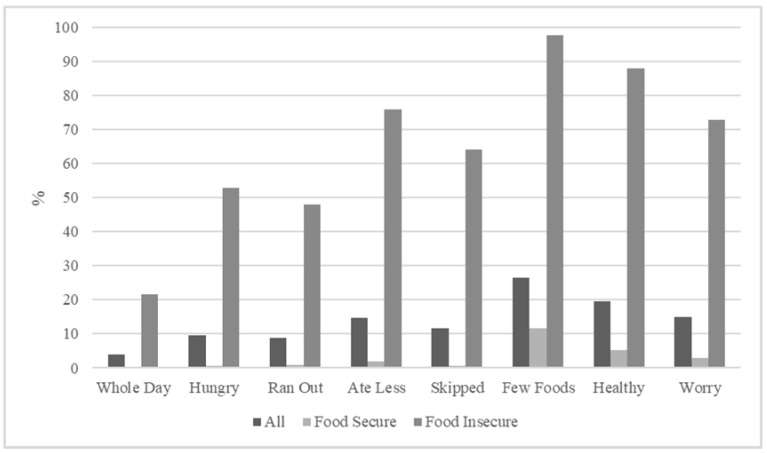
Affirmative responses (%) to the FIES questions (items).

Referring to the last 12 months, Figure 1 shows respondents answering “yes” for the food security indicators [19]. Of the food security indicators, consuming few kinds of food was the most common when dealing with periods of FI (26.4%), and 3.9% went a whole day without eating due to a lack of food or other resources. Among food-insecure respondents, consuming few kinds of foods had the most affirmative answers (97.6%), followed by having the inability to eat healthfully (88%) and consuming less than required (76%) due to a lack of money or resources. Food-secure respondents also experienced some of the indicators. Those experiencing food security responded affirmatively to consuming few kinds of foods (11.6%), being unable to eat healthfully (5.3%), and experiencing worry they might not have enough food to eat (3%) due to a lack of money or resources; see Figure 1.

### 3.3. FI-Related Factors during the COVID-19 Pandemic

Table 3 shows the differences in academic performance, in the amount of food consumed, and in support circle by food security status. Regarding academic performance, well over half of the food-insecure respondents were affected in a negative way (64.5%) compared to 21% affected in a positive way. Under half of the food-secure respondents reported negative changes in academic performance (48%) compared to 23.8% who reported positive changes. When looking at changes in food consumption among food-insecure respondents, 50% consumed less compared to 12.7% who consumed more. Among food-secure respondents, 21.5% consumed more, while 14% consumed less. Most food-secure respondents, or over half, consumed about the same (61.5%). Regarding respondents’ support circle, 9% of food-insecure respondents had a large circle of family and friends to help them. Within the food-secure group, 37.7% of respondents had a large support circle.

After adjusting for gender and age, food-secure respondents were found to be less likely to have negative academic performance compared to food-insecure respondents (OR = 0.53, 95% CI [0.29, 0.91], *p* = 0.02). Secondly, food-secure respondents had nearly twice the likelihood of experiencing positive or neutral academic performance compared to food-insecure respondents (OR = 1.94, 95% CI [1.09, 3.52], *p* = 0.02).

The results suggest that food security status is significantly associated with changes in food consumption with food-secure respondents being significantly less likely to report decreased food consumption (OR = 0.14, 95% CI [0.08, 0.26], *p* < 0.0001) and a nearly four times higher likelihood of maintaining the amount of food consumed (OR = 3.92, 95% CI [2.14, 7.18], *p* < 0.0001) compared to food-insecure respondents.

Respondents who reported having a large support network were nearly five times more likely to be food-secure compared to those who were experiencing FI (OR = 4.94, 95% CI [2.04, 12.0], *p* < 0.0005).

## 4. Discussion

The purpose of the present study was to assess the prevalence of FI and evaluate possible related factors among university students in Iceland during the COVID-19 pandemic. We found that nearly one in six respondents experienced FI during the pandemic. The high rates of FI reported in the current study are related to a lack of social support, a decrease in the amount of food eaten, and negative changes in academic performance.

While not as high as in colleges and universities in other developed countries, for example, 33% to 41% in the US and 19–63% seen in other research [5,6,7,8,25], this survey still found that about 17% of university students in Iceland, aged 18 and older, are currently living with some level of FI, after removing missing responses. About 4% of respondents (*n* = 28) reported going an entire day without eating due to a lack of money or other resources to acquire food, referring to the last 12 months.

Examples of coping strategies for individuals experiencing FI include purchasing lower-cost items and less nutritious items, for example, lower micronutrients, protein, and dietary fiber; consuming a limited variety of foods or the same few foods more often; and increased cognitive attention towards obtaining food. Instances of increased cognitive attention to obtaining food include a preoccupation or fixation with where the next meal will come from and food stress, anxiety, and fear [26]. As seen in this study, university students in Iceland experiencing FI also utilize some coping techniques such as eating few types of foods (97.6%, *n* = 122), skipping meals (64.0%, *n* = 80), and eating less than required or desired (76.0%, *n* = 95) (*p* < 0.001). Based on these results, there is a noticeable disparity in the responses from food-insecure and food-secure students, indicating a significant association between food security status and the amount of food consumed by students, suggesting that food security status is a key determinant of the amount of food consumed among students.

University students may be affected by FI and, therefore, the factors that often come along with it, such as hunger; mental health issues, including depression and the inability to concentrate; and lower academic achievement. FI can also have a negative impact on university students’ overall health, including malnutrition and immune function [11,27]. As revealed in this study, university students in Iceland experiencing FI are also susceptible to adverse effects on academic performance. Compared to food-insecure students, food-secure students were significantly less likely to have their academic performance affected negatively by COVID-19.

When looking deeper at students’ academic experiences or feelings in other countries, Maroto et al. found that food-insecure students were more likely to report a lower grade point average (GPA) than a high GPA compared to food-secure classmates [28]. While this does not prove that FI always causes poorer academic performance, it adds to the growing body of evidence suggesting an association between it and poorer academic performance [28,29].

A financial coping strategy, especially for young adults at university, is to lean on their support circle of friends and family. Having a large support network of family or friends can play an enormous role in the health outcomes of food-insecure individuals [30,31]. According to the responses in the current survey, when compared to food-insecure respondents, food-secure respondents were nearly five times more likely to have a large support network, which enables food-secure students to lean on family and friends more often when in need of support.

This study is strengthened as it broadly represents the student population, as seen in the ratio between male and female students at the University of Iceland, approximately 32% and 68% in 2022, respectively [32]. According to Statistics Iceland, in 2022, 54.6% of university students in Iceland were aged 26 and above, similar to our respondents’ age distribution [4].

The study does well to provide an important look into the circumstances among university students. Additionally, using the FIES to measure food security is a validated, accurate, and direct measure. The limitations of this study include the length of recall time of 12 months for the food security situation, which may add recall bias to responses, and a low response rate of about 4.4%, possibly due to the need for more advertising. In any case, similarly, low response rates have been reported by other investigators assessing FI prevalence in college and university students [8].

Additionally, responses from students who skipped one or more food security indicator question were given a value of zero. Therefore, it is not possible to say definitively if those respondents were or were not food-secure. For academic changes, it was not specified what qualifies as negative or positive, nor did it ask what the negative or positive circumstances were. It is important to note that this study employed a cross-sectional design, meaning it captured data at a single point in time. Therefore, while it provides valuable insights into the prevalence of food insecurity, it cannot establish causality between the observed prevalence and the COVID-19 pandemic alone. Survey respondents could choose whether to participate, which may have introduced selection bias.

## 5. Conclusions

The results from this Food Insecurity among European University Students during the COVID-19 Pandemic (FINESCOP) study reveal that FI does affect university students in Iceland, and some factors, such as social support networks and negative changes in academic performance, are related to this FI. Future food assistance programs should be highlighted to keep students out of FI and prevent anyone from having to go an entire day without eating due to a lack of money or other resources.

In Iceland, there is limited published research on food security at the individual level, which can lead to a lack of assistance, public health programs, and policy creation for specific groups, such as university students. Additionally, there are few to no specific studies or data on university students and their food security status in Iceland. This leaves the personal views, experiences, and behaviors of food-insecure students under-represented, which can have significant implications for nutritional health outcomes for individuals experiencing FI or hunger and the policies that can be created to protect them.

## Figures and Tables

**Table 1:** Respondent characteristics.

Variables	*n*	%
Age		
18–25	147	15.9
26–35	210	22.7
36+	151	16.3
Missing	416	45.0
Gender		
Female	688	74.5
Male	199	21.5
Non-binary	14	1.5
Other ^ab^	6	0.6
Missing	17	1.8
Origin		
Iceland	725	78.5
Europe, other than Iceland	125	13.5
Americas (North, South, and Central)	30	3.2
Africa, Asia, Oceana	22	2.3
Prefer not to say	3	0.3
Missing	19	2.1
Location in Iceland		
Capital region ^c^	694	75.1
Other ^ad^	102	11
Prefer not to say	8	0.9
Missing	120	13
Employment before COVID-19		
Employed full-time	284	30.7
Employed part-time	313	33.9
Self-employed	15	1.6
Not employed but looking for a job	20	2.2
Not employed NOR looking for a job	114	12.3
Other ^ab^	54	5.8
Missing	124	13.4
Parent/Guardian education		
No education or primary education ^ae^	41	4.4
Lower and upper secondary education ^a^	182	19.7
Post-secondary, non-tertiary	87	9.4
First stage of tertiary (BSc or BA)	245	26.5
Second stage of tertiary (MSc or Ph.D.)	313	33.9
Do not want to answer	19	2.1
Missing	37	4

^a^ Combined due to few responses. ^b^ Includes responses “other”, “prefer not to say”, “do not want to answer”, or “unsure”. ^c^ “Capital region” includes Reykjavík, Kópavogur, Hafnarfjörður, Garðabær, Mosfellsbær, Seltjarnarnes, Kjósarhreppur. ^d^ “Other” includes Western Region, Westfjörds, Northwest Region, Northeast Region, East Region, South Region, Reykjanes Peninsula. ^e^ Responses include “no education”, “pre-primary education”, and “primary education”.

**Table 2:** Respondents’ educational characteristics.

Variables	*n*	%
Current level of studies		
Undergraduate	491	53.1
Postgraduate	356	38.5
Neither undergraduate nor postgraduate	37	4
Missing	40	4.3
Current year of studies		
First academic year	261	28.2
Second academic year	268	29
Third academic year	160	17.3
Fourth, fifth, or sixth academic year ^a^	109	11.8
Other	47	5.1
Missing	79	8.5
Current field of study		
Agriculture, forestry, fisheries, and veterinary	5	0.5
Arts and humanities	94	10.2
Business, administration, and law	94	10.2
Education	98	10.6
Engineering, manufacturing, and construction	41	4.4
Health and wellness	170	18.4
Information and communication technologies	24	2.6
Natural sciences, mathematics, and statistics	65	7
Service management	2	0.2
Social sciences, journalism, and information	95	10.3
Other ^ab^	144	15.6
Missing	92	10.1
Type of teaching received		
Blended (face-to-face and online)	471	51
Face-to-face on campus	41	4.4
No teaching received	58	6.3
Virtual or online	257	27.8
Missing	97	10.5

^a^ Combined due to few responses. ^b^ Includes responses “other”, “prefer not to say”, “do not want to answer”, or “unsure”.

**Table 3:** Changes in academic performance, amount of food consumed, and support circle by food security status.

Variables ^b^	Food-Insecure ^a^ *n* = 125		Food-Secure *n* = 602		*p*-Value
	*n*	%	*n*	%	
Changes in academic performance					**<0.05**
In a negative way (very and somewhat)	80	64.5	288	48.0	
In a positive way (very and somewhat)	26	21.0	143	23.8	
Not affected	13	10.5	137	22.8	
Do not know or prefer not to say	5	4.0	32	5.3	
Changes in the amount of food consumed					**<0.001**
Consumed more	14	12.7	118	21.5	
Consumed about the same	34	30.9	338	61.5	
Consumed less	55	50.0	77	14.0	
Do not know	7	6.4	17	3.1	
Support circle					**<0.001**
I have a large support circle of family and friends to help me	9	9.0	184	37.7	
I have neither friends nor family to support me	7	7.0	21	4.3	
I have some family and some friends to support me	29	29.0	119	24.4	
I only have some family to support me	37	37.0	102	20.9	
I only have some friends to support me	16	16.0	41	8.4	
Do not want to answer	2	2.0	21	4.3	

^a^ Food insecurity status according to the criteria of the modified version of the USDA’s scoring system [23]. ^b^ Chi-squared test of independence for categorical variables; *p*-values are significant in bold (*p* < 0.05).

## Data Availability

Data will be provided upon request to the corresponding author. A formal data-sharing agreement must be signed before data are released. The data are not publicly available due to ongoing processing for comparison with all participating countries, pending publication in an article.
